# Evaluation of Motion Artifact Correction Technique for Cone-Beam Computed Tomography Image Considering Blood Vessel Geometry

**DOI:** 10.3390/jcm13082253

**Published:** 2024-04-12

**Authors:** Yunsub Jung, Ho Lee, Hoyong Jun, Soobuem Cho

**Affiliations:** 1 Department of Materials and Production, Aalborg University, 9220 Aalborg East, Denmark; yunsubjung9909@gmail.com; 2 Department of Radiation Oncology, Yonsei Cancer Center, Heavy Ion Therapy Research Institute, Yonsei University College of Medicine, Seoul 03722, Republic of Korea; holee@yuhs.ac; 3 Department of Radiology, College of Medicine, Ewha Womans University, Seoul 03760, Republic of Korea; kingnose80@gmail.com

**Keywords:** CBCT, motion artifact correction, motion-corrected index, vessel analysis, visual grading analysis, transcatheter chemoembolization

## Abstract

**Background:** In this study, we present a quantitative method to evaluate the motion artifact correction (MAC) technique through the morphological analysis of blood vessels in the images before and after MAC. **Methods:** Cone-beam computed tomography (CBCT) scans of 37 patients who underwent transcatheter chemoembolization were obtained, and images were reconstructed with and without the MAC technique. First, two interventional radiologists selected the blood vessels corrected by MAC. We devised a motion-corrected index (MCI) metric that analyzed the morphology of blood vessels in 3D space using information on the centerline of blood vessels, and the blood vessels selected by the interventional radiologists were quantitatively evaluated using MCI. In addition, these blood vessels were qualitatively evaluated by two interventional radiologists. To validate the effectiveness of the devised MCI, we compared the MCI values in a blood vessel corrected by MAC and one non-corrected by MAC. **Results:** The visual evaluation revealed that motion correction was found in the images of 23 of 37 patients (62.2%), and a performance evaluation of MAC was performed with 54 blood vessels in 23 patients. The visual grading analysis score was 1.56 ± 0.57 (radiologist 1) and 1.56 ± 0.63 (radiologist 2), and the proposed MCI was 0.67 ± 0.11, indicating that the vascular morphology was well corrected by the MAC. **Conclusions:** We verified that our proposed method is useful for evaluating the MAC technique of CBCT, and the MAC technique can correct the blood vessels distorted by the patient’s movement and respiration.

## 1. Introduction

Cone-beam computed tomography (CBCT), which is widely used in interventional procedures, provides important guidelines for identifying abnormal structures of blood vessels and suggesting interventional procedures and surgeries in a non-invasive manner [1]. CBCT requires a relatively long scan time compared to general computed tomography (CT) and generally requires the patient to stop breathing for at least 5 s. However, for elderly patients or those with respiratory failure, holding their breath is a difficult process. Therefore, CBCT images of patients often have poor image quality because of motion artifacts due to the patient’s movement or respiration [2,3]. Motion artifacts caused by the patient’s movement include image blurring and the severe distortion of the shape of blood vessels [4]. Since the blood vessels in CBCT images are the most important structures for interventional procedures, additional CBCT scans may be required if the vessel shape is severely distorted.

There is no definitive method for eliminating breathing-induced motion artifacts in CBCT images. The most practical way is for the radiographer to practice a breathing routine that is good at inducing the patient to stop breathing [3]. Although breathing control is a practical way to reduce the occurrence of significant motion artifacts, it cannot fundamentally eliminate the occurrence of motion artifacts. Therefore, various post-processing techniques (motion artifact correction, MAC) have been developed and commercialized to correct distorted blood vessels due to involuntary respiratory movements [5,6,7,8,9,10,11]. Additionally, as CBCT equipped with MAC technique has become widely available, several studies evaluating their performance have been published [8,9,10,12,13,14,15,16]. Image distortion due to respiratory motion was corrected using the patient-specific respiratory motion model, and the displacement by motion was measured to evaluate the MAC [9]. Uncorrected CBCT and respiratory motion-corrected CBCT were compared with respiration-gated CBCT. They used the normalized cross-correlation cost function to measure the pixel similarity of images before and after MAC [12]. Head movement was calculated using the cross-correlation between every two successive projection images, and motion artifacts were corrected using this motion information. They quantitatively compared the images before and after MAC using the structural similarity index and root-mean-square error, which are image evaluation metrics [10]. Images from CT scans of the coronary artery of patients with a high heart rate were evaluated before and after MAC. Blood vessel eccentricity was measured on cross-sectional images of blood vessels and compared with subjective evaluation by a radiologist [13]. The anthropomorphic dynamic heart phantom underwent CT to evaluate the motion compensation performance. Measurement accuracy was evaluated by measuring the measurement errors of the minimal luminal diameter and minimal luminal area in the cross-sectional area of the coronary artery inside the heart phantom [14]. CT attenuation values inside the coronary artery were measured using CT images of free-breathing patients [15]. To evaluate MAC, maximum intensity, sharpness, and full width at half maximum were measured in segmental hepatic artery vessels using maximum intensity projection (MIP) images [16].

Existing qualitative evaluations of MAC techniques were performed by visually evaluating image 2D cross-sections or 3D reconstructed images before and after MAC [13,14,16] or by presenting the diagnostic accuracy of radiologists [13,15]. However, most quantitative evaluation studies were conducted using various quantitative metrics, but in most cases, the evaluation target was limited to 2D images. These studies measured the degree of change in areas such as blood vessels in 2D images [9,10,12,13,14,15,16]. In cases where 2D images are mainly used, such as diagnostic CT, it is also meaningful to evaluate 2D-based image quality. However, in the case of CBCT for interventional procedures, it is important to visualize human organs as 3D [17]. Additionally, evaluation methods for the 3D shape of the structure rather than the entire image are needed to evaluate algorithms that selectively correct specific structures such as blood vessels [18].

Another important factor to evaluate MAC is connectivity. In the case of thin and long structures such as blood vessels, the shape of the structure is interrupted due to movement artifacts. The structural similarity (SSIM) [19], which calculates the difference from the comparison image, and full width at half maximum (FWHM) [20], which evaluates the edge characteristics of the structure, are good metrics for evaluating the effectiveness of MAC and have been used in studies evaluating the performance of MAC [16,21,22]. However, since these metrics only compute the difference from the comparison image (full-reference metric), they cannot measure the connectivity of the structure.

In this study, we propose a new quantitative method and metric to evaluate MAC techniques in CBCT images. First, the morphological information of blood vessels existing in 3D space is projected into 2D space, and then the images before and after MCA are quantitatively evaluated based on the blood vessel information. To verify the proposed method, we compared our method in areas where motion artifacts were corrected and areas where motion artifacts were not corrected. Additionally, the proposed quantitative method was compared with qualitative evaluation by interventional radiologists.

## 2. Materials and Methods

### 2.1. Data Acquisition

**Figure 1:**
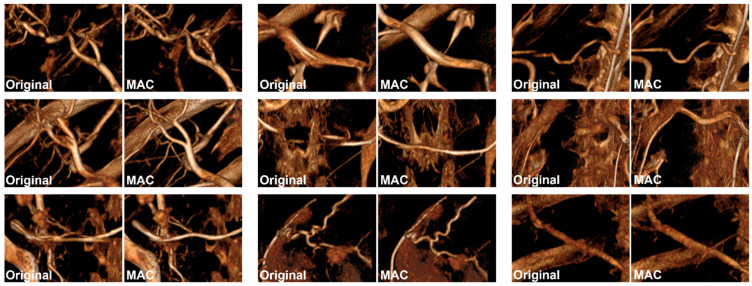
3D rendered images with original (**left**) and motion artifact correction (MAC) applied (**right**).

This study was approved by the institutional review board, and the CBCT (XA; GE Healthcare, Chicago, IL, USA) images of 37 patients who underwent transcatheter chemoembolization were used. A non-ionic contrast agent (Unihexol 300; Union Korea Pharm, Seongnam-si, Republic of Korea or Scanlux 300; Sankem Healthcare, Inc., Metro Manila, Philippines) was injected into the common hepatic artery at a rate of 4~5 mL per second. After the contrast agent was injected for approximately 5 s, the CBCT scan was started. Contrast agent injection continued during the CBCT scan. CBCT scan time took an average of 6 s. This total 10~11 s procedure was taken in place without holding breath. For each patient, images with and without the MAC technique were reconstructed (Figure 1). We performed MAC using the function installed in the CBCT equipment (Motion Freeze; GE Healthcare, Chicago, IL, USA). The resulting image was reconstructed with the axial thickness (0.454 mm) and image size (512 × 512).

### 2.2. Motion-Corrected Index

**Figure 2:**
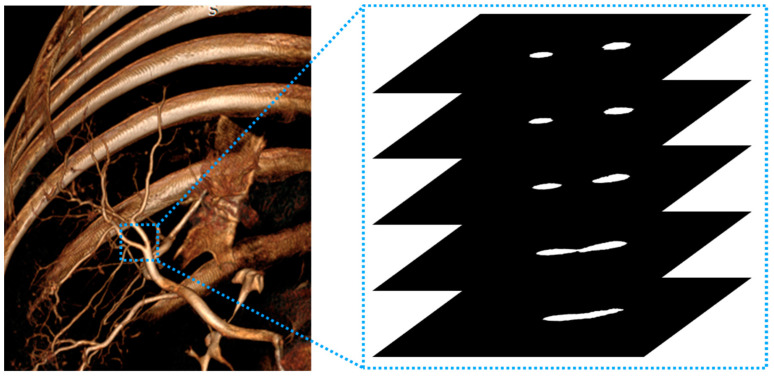
Rendered blood vessel shape in 3D space (**left**) and 2D cross-section (**right**) of blood vessel area. The 2D axial images show the results of selectively segmenting only blood vessels.

In our preliminary visual inspection test, it was confirmed that “MAC only corrects the artifacts of blood vessels with a certain diameter or higher”. Therefore, a metric was needed to quantify the performance of MAC based on blood vessel information. Blood vessels, which are 3D structures, only have information about a partial cross-section in a 2D image (Figure 2). In our preliminary tests, previously published metrics were methods based on 2D image information and, therefore, did not reflect the effect of MAC on 3D structures. Therefore, we needed a method to reflect information in 3D space into a single 2D image. Additionally, we designed a full-reference-based metric [23] that compares images after MAC with the images before MAC because there is no absolute index for the effect of MAC.

**Figure 3:**
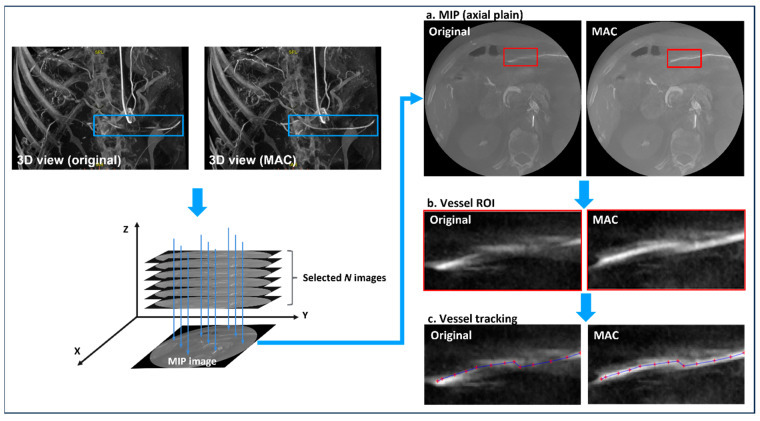
A schematic diagram of the proposed algorithm for quantitative evaluation of motion artifact correction (MAC). Maximum intensity projection (MIP) images are reconstructed using multiple axial images (blue box) including blood vessel regions manually selected by an intervention radiologist. The blood vessel region of interest (ROI) for analysis is extracted from the MIP image (red box), and the centerline is manually drawn from the extracted vessel. Pixel information on the obtained centerline is extracted from the original image and the MAC-applied image, respectively.

**Figure 4:**
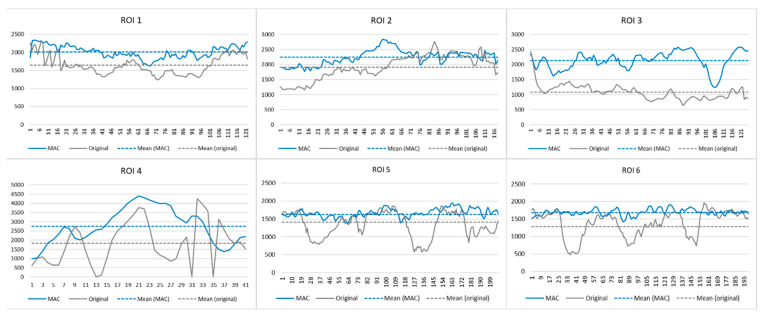
The profile for a vascular centerline was selected by an interventional radiologist. The pixel values constituting the centerline of the blood vessel are extracted from the same position of the original image and the image to which motion artifact correction (MAC) is applied. The *x*-axis shows the start and end of the centerline inside the blood vessel, and the *y*-axis shows the pixel values in the maximum intensity projection image.

An algorithm is designed for the morphological analysis of vascular areas before and after MAC (Figure 3). First, the vascular region for evaluation was selected from the before-and-after MAC images. This region consists of a set of several 2D axial images. A maximum intensity projection (MIP) image is reconstructed [24] using all axial images including the selected blood vessel region (a). Then, the region of interest (ROI) including the blood vessel was manually extracted from the MIP image (b). A vessel without other structures around it was selected when extracting the vascular ROI from the MIP image. An interventional radiologist manually marked the centerline of a vessel on an ROI image (c). A profile was constructed using pixel values forming the centerline of the blood vessel (Figure 4). Then, pixel values (Equations (1) and (2)) of the centerline were extracted from the original (non-corrected) and motion-corrected profiles, respectively.

(1) $$P_{nc}=\left[p_{1}^{nc},p_{2}^{nc},p_{3}^{nc},\ldots ,p_{k}^{nc}\right]$$

(2) $$P_{c}=\left[p_{1}^{c},p_{2}^{c},p_{3}^{c},\ldots ,p_{k}^{c}\right]$$

where $P_{nc}$ and $P_{c}$ matrices represent the MIP pixel values of the profile obtained from the uncorrected original images and motion-corrected images, respectively. Also, *k* represents the pixel number of arrays constituting the centerline drawn inside the blood vessel. The extraction process of the pixel value was performed at the same location in both MIP images. Each extracted matrix in the uncorrected original and motion-corrected images is an index that reflects the pixel value inside the blood vessel and is used in the following formula:

(3) $$MCI=\frac{\sum_{i=1}^{k}\left(P_{i}^{c}-P_{i}^{nc}\right)}{\sum_{i=1}^{k}\left(P_{i}^{nc}\right)}\times \frac{\sigma_{nc}}{\sigma_{c}}$$

The motion-corrected index (MCI) is a metric that shows the difference between motion-corrected and uncorrected original images (Equation (3)). Here, $\sigma_{nc}$ and $\sigma_{c}$ represent the standard deviations of the uncorrected original and motion-corrected images, respectively. The better the motion is corrected for by MAC, the brighter the blood vessel regions appear in the MIP image. Therefore, since the profile value of $P_{c}$ is higher than $P_{nc}$, there is no limit to the maximum value of MCI. The larger the value of MCI, the better the MAC, and the closer it is to 0, the less the correction.

### 2.3. Experiment 1

Experiment 1 was performed to identify blood vessels in which motion artifacts were corrected through visual evaluation by an interventional radiologist. An interventional radiologist evaluated paired CBCT images before and after MAC. In each patient’s images, interventional radiologists detected areas of blood vessels whose morphology had improved due to MAC. This experiment was conducted using a total of 74 cases for 37 patients. This evaluation was performed by one interventional radiologist with 7 years of experience in interventional radiology.

### 2.4. Experiment 2

Experiment 2 qualitatively and quantitatively evaluated the MAC technique through the morphological evaluation of blood vessel ROI found in Experiment 1. The evaluators were provided with a pair of original images and an artifact-corrected image, and we asked the evaluators to compare the artifact-corrected image to the original image as a reference. The evaluation was conducted using 54 ROIs extracted from 23 patients. For qualitative evaluation, we used a 5-point scale visual grading analysis (VGA) method [25]. The 5-point scale was rated as follows: grade +2 (clearly better than the reference image), grade +1 (slightly better than the reference image), grade 0 (equal to the reference image), grade −1 (slightly inferior to the reference image), and grade −2 (clearly inferior to the reference image). Visual evaluation was performed by two interventional radiologists with 3 and 7 years of experience in interventional radiology, respectively. In addition, we computed the MCI from 54 ROIs evaluated by the evaluators.

### 2.5. Experiment 3

To validate the devised MCI metric, we compared the MCI values in MAC-corrected vessels and MAC-uncorrected vessels. In addition to the 54 vascular ROIs in which the artifacts were corrected in Experiment 2, the MCI was calculated in 10 ROIs in which motion artifacts were not corrected. A total of 54 ROIs are vascular regions identified by interventional radiologists as having good motion artifact correction, and 10 ROIs are randomly selected regions from the uncorrected region after the MAC.

### 2.6. Statistical Analysis

The Wilcoxon signed-rank test was used for the quantitative evaluation of images before and after MAC. All statistical analyses were performed using statistical software (SPSS, version 22.0; IBM Corp., Armonk, NY, USA), and *p* values of <0.05 were considered statistically significant. The quantitative evaluation method proposed in this study was implemented using MATLAB (2019a; MathWorks, Natick, MA, USA).

## 3. Results

Table 1 shows the results of visual evaluation by interventional radiologists (Experiment 1). Among the 37 patients’ data used for evaluation, the blood vessels were corrected using MAC in 23 patients (62.2%). In the images of the remaining 14 patients (37.8%), the distortion of blood vessels due to movement was not confirmed. The areas corrected through the MAC technique were identified as (1) contrast-enhanced, (2) blood vessel shape severely distorted due to respiratory artifacts, and (3) blood vessel area over a certain thickness (approximately 13 mm). 

Experiment 2 was performed using 54 ROIs obtained from the 23 patients’ data found in Experiment 1, the results of which are summarized in Table 2. Comparing the corrected CBCT images with the uncorrected images, radiologist 1 evaluated grade +2 in 32 ROIs (59.26%, *p* < 0.001), grade +1 in 20 ROIs (37.04%, *p* < 0.001), and grade 0 in 2 ROIs (3.70%, *p* < 0.001). Radiologist 2 was evaluated as grade +2 in 34 ROIs (62.96%, *p* < 0.001), grade +1 in 16 ROIs (29.63%, *p* < 0.001), and grade 0 in 4 ROIs (7.41%, *p* < 0.001). The mean and standard deviation of the VGA scores of the radiologists were similar, with radiologist 1 scoring 1.56 ± 0.57 and radiologist 2 scoring 1.56 ± 0.63. The proposed MCI value calculated for the same 54 ROIs scored 0.67 ± 0.11 (min. 0.40 ~ max. 0.91).

In Experiment 3, the MCI of the uncorrected blood vessels by MAC was calculated as 0.0035 ± 0.0088 (Table 3), which was close to 0 compared to the MCI (mean = 0.67) of the 54 blood vessels corrected for motion artifact. Experiment 3 was a test to verify the designed method, and the MCI showed the performance of MAC well.

## 4. Discussion

With the increasing number of catheter interventions, planning and implementing procedures using CBCT has become an important issue [26]. Underqualified CBCT images result in additional image acquisition for exact targeting and catheterization. This requires additional radiation exposure to both patients and radiologists and is time-consuming for radiologists [27]. However, the acquisition of qualified CBCT images can lead to a more accurate diagnosis and appropriate treatment. Therefore, various attempts are being made to reduce distortion caused by the patient’s movement and respiration and improve the quality of CBCT images [28,29,30,31,32,33]. CBCT images are very important images that provide direct information for catheter intervention. Therefore, the objective verification of new functions for image improvement is required. For this verification, a qualitative evaluation using quantitative metrics must be performed along with a qualitative evaluation through the eyes of interventional radiologists.

In this study, a quantitative method was presented to evaluate the qualitative improvement of the MAC technique to correct the distortion of blood vessels. As there is no existing method to evaluate the motion compensation of a structure in a 3D space, a new method was proposed for this purpose. We verified the MAC technique of the CBCT equipment using the proposed quantitative and qualitative evaluation methods by an interventional radiologist. Thus, it was confirmed that the MAC technique of the CBCT equipment could correct the blood vessels distorted by the patient’s respiration and movement. The evaluation results obtained using the proposed quantitative evaluation method were verified together with the qualitative results evaluated by an interventional radiologist. Thus, it was confirmed that the motion compensation function of the CBCT equipment can correct the blood vessels distorted by the patient’s movement and respiration. In addition, to verify the MCI metric presented in this study, blood vessels corrected by the MAC technique and blood vessels that were not corrected were evaluated. The evaluation confirmed that our proposed MCI is a metric that correctly evaluates the degree of MAC.

Our study has several limitations. First, we did not identify quantitative figures for the conditions under which the MAC technique was applied. In Experiment 1, we visually confirmed that the MAC technique only corrected the blood vessels under specific conditions (diameter, degree of distortion, and contrast enhancement); however, we could not determine a specific numerical value for this. A study using a moving phantom with imitated human blood vessels is required to accurately evaluate the vascular conditions to which the MAC technique is applied. Second, we did not evaluate the adverse effects of the MAC technique on image quality. It is necessary to consider cases in which structures other than blood vessels are distorted by the MAC technique. Third, we did not derive a correlation coefficient between the qualitative and quantitative evaluations. Because the 5-point scale VGA method we used for qualitative evaluation has five discrete values without intermediate steps and the MCI metric we presented is a continuous value, the correlation coefficient between these two indicators will have a large error.

## 5. Conclusions

A technique to correct motion artifacts caused by patient breathing and movement in CBCT scans has been commercialized. The qualitative evaluation of this technique has been studied through radiologist visual inspection, but there are no existing methods to quantitatively validate it. We presented a new method to evaluate the effectiveness of MAC in 3D space and compared it with the qualitative results of radiologists. By comparing the qualitative evaluation of the method presented in this study, we demonstrated that our method is suitable for MAC evaluation. In addition, through our new and qualitative methods, it was confirmed that the MAC technique of the CBCT equipment is useful in correcting artifacts caused by patient movement and breathing.

## Figures and Tables

**Table 1:** Results of Experiment 1.

Patient	1	2	3	4	5	6	7	8	9	10	11	12	13
MAC	C	UC	C	UC	C	UC	UC	C	C	C	C	C	C
Num. of ROI *	1	None	4	None	1	None	None	1	5	1	3	1	1
Patient	14	15	16	17	18	19	20	21	22	23	24	25	26
MAC	C	UC	C	C	C	UC	C	C	C	C	UC	UC	C
Num. of ROI	3	None	3	2	3	None	5	3	3	1	None	None	2
Patient	27	28	29	30	31	32	33	34	35	36	37	-	-
MAC	C	C	UC	UC	UC	UC	C	C	UC	UC	C	-	-
Num. of ROI	2	3	None	None	None	None	1	3	None	None	2	-	-

Note: The number of corrected vascular found by an interventionist radiologist. MAC: motion artifact correction, C: corrected, UC: uncorrected, ROI: region of interest. * Number of regions in which MAC was found.

**Table 2:** Results of Experiment 2.

ROI	1	2	3	4	5	6	7	8	9	10	11	12	13	14
VGA (R1)	2	1	2	2	2	1	2	0	1	2	1	2	2	1
VGA (R2)	2	1	2	2	2	2	2	1	0	2	2	2	2	0
MCI *	0.426	0.402	0.782	0.628	0.712	0.865	0.827	0.912	0.534	0.548	0.812	0.485	0.646	0.817
ROI	15	16	17	18	19	20	21	22	23	24	25	26	27	28
VGA (R1)	1	1	1	2	2	1	2	1	0	2	1	2	2	2
VGA (R2)	1	1	1	2	2	2	2	1	1	1	1	1	2	2
MCI	0.698	0.751	0.675	0.586	0.705	0.712	0.531	0.794	0.912	0.698	0.698	0.582	0.575	0.586
ROI	29	30	31	32	33	34	35	36	37	38	39	40	41	42
VGA (R1)	2	2	1	1	2	2	2	1	1	2	1	2	2	2
VGA (R2)	2	2	2	1	2	2	2	1	0	2	1	2	2	2
MCI	0.711	0.548	0.651	0.742	0.531	0.648	0.705	0.771	0.802	0.641	0.698	0.658	0.575	0.546
ROI	43	44	45	46	47	48	49	50	51	52	53	54	-	-
VGA (R1)	2	1	1	2	2	2	2	1	2	1	2	2	-	-
VGA (R2)	2	2	1	2	1	2	2	0	1	2	2	2	-	-
MCI	0.752	0.711	0.695	0.652	0.512	0.623	0.721	0.771	0.692	0.755	0.646	0.511	-	-

Note: VGA is a qualitative evaluation by two radiologists, and the corrected value is a metric proposed in this study for quantitative evaluation. MCI: motion-corrected index, R: radiologist, ROI: region of interest, VGA: visual grading analysis. * A value closer to 0 indicates less motion artifact correction.

**Table 3:** Results of Experiment 3.

	Uncorrected											Corrected ^†^
ROI	1	2	3	4	5	6	7	8	9	10	Mean	Mean
MCI *	−0.008	0.007	0.006	0.015	−0.002	0.009	−0.013	0.002	0.008	0.011	0.004	0.67

MCI: motion-corrected index, ROI: region of interest. * A value closer to 0 indicates less motion artifact correction. ^†^ Mean of the MCI value computed using 54 blood vessels corrected by MAC.

## Data Availability

The data presented in this study are available on request from the corresponding author due to privacy and legal restrictions.
